# miR-33a-5p inhibits the progression of esophageal cancer through the DKK1-mediated Wnt/β-catenin pathway

**DOI:** 10.18632/aging.203430

**Published:** 2021-08-23

**Authors:** Qingping Song, Hui Liu, Chengyan Li, Haifeng Liang

**Affiliations:** 1 Department of Surgery, Tumor Hospital of Liaocheng, Liaocheng 252000, Shandong, China; 2 Department of Gastroenterology, Yantai Affiliated Hospital of Binzhou Medical University, Yantai 264000, Shandong, China

**Keywords:** miR-33a-5p, DKK1, esophageal cancer, Wnt/β-catenin, progression

## Abstract

Esophageal cancer (EC) is one of the most lethal malignancies in humans, and multiple miRNAs have been identified to modulate EC progression by targeting different targets. However, the effect and related mechanism of microRNA-33a-5p (miR-33a-5p) on EC development remain elusive. In this study, we explored the clinical value, function, and possible mechanism of miR-33a-5p in EC. We uncovered that miR-33a-5p and DKK1 are involved in the progression of EC. Significantly, the expression levels of miR-33a-5p were reduced and DKK1 levels were elevated in serum and tissues of clinical EC samples and in EC cell lines. The downregulation of miR-33a-5p and DKK1 upregulation were related to high TNM staging and poor differentiation of patients. The area under the curves (AUCs) of miR-33a-5p and DKK1 for the occurrence of EC were 0.914 and 0.900, respectively. Down-regulation of miR-33a-5p or overexpression of DKK1 indicated a worse prognosis. The miR-33a-5p overexpression or DKK1 depletion induced apoptosis and repressed proliferation, migration, and invasion of EC cells. The repression of miR-33a-5p by inhibitor or DKK1 overexpression presented the conversed effects on EC cells. Mechanically, miR-33a-5p suppressed DKK1 expression, and miR-33a-5p targeted DKK1 to affect the biological behavior of EC through the Wnt/β-catenin pathway. Meanwhile, miR-33a-5p inhibited the tumor growth of EC *in vivo*. Thus, we concluded that miR-33a-5p inhibited the progression of EC through the DKK1-mediated Wnt/β-catenin pathway. MiR-33a-5p and DKK1 can be used as potential therapeutic targets of EC.

## INTRODUCTION

Esophageal cancer (EC), which accounts for about 2% of human malignancies, is one of the most lethal malignancies in humans [1]. Among the subtypes of EC, esophageal adenocarcinoma mainly occurs in European and American countries, while in China, esophageal squamous cell carcinoma (ESCC) is the dominant histologic subtype [2]. The lesions of early EC are limited to the mucosa and submucosa, which are not easy to be detected in early stage because routine endoscopic examination often cannot clearly see the changes in the submucosal structure of esophagus. As a result, it often progresses to advanced EC characterized by high invasion and metastasis, high postoperative mortality and poor prognosis [3]. Besides, the occurrence and development of EC is a very complex process, involving a variety of carcinogenic signal transduction pathways [4]. Therefore, understanding the possible molecular mechanism of the occurrence and progression of EC may contribute to the prevention and treatment of the disease.

microRNAs (miRNAs), which are expressed in almost all human cancers, act as tumor suppressors or oncogenes and regulate gene expression by specific binding to the 3′ non-translated region of downstream target genes [5, 6]. It was previously confirmed that miR-33a-5p was under-regulated in solid tumors, including hepatocellular carcinoma [7], osteosarcoma [8], and prostate cancer [9]. Zhang et al. [10] reported that miR-33a-5p was low expressed in ESCC cells and tissues, and can inhibit the progression of ESCC by regulating DANCR/axis. However, its specific clinical value and molecular mechanisms in EC remain to be explored. In this study, we predicted through database prediction that DKK1 was a potential target of miR-33a-5p. As to DKK1, it can regulate signal transduction in cells by binding to corresponding receptors, thus determining the characteristics of cell proliferation and differentiation [11]. There are different reports on the role of DKK1 in tumors. It is highly expressed in liver cancer [12] and stomach cancer [13], and is a regulator of the Wnt signaling pathway. The Wnt/β-catenin signaling pathway is the most important and well-studied signaling pathway in the Wnt pathway, which is closely related to the occurrence and development of many solid tumors [14]. Studies have pointed out that miR-590-3p can directly target DKK1 to promote the progression of colon cancer cells through the Wnt/β-catenin pathway [15]. However, the molecular mechanism of miR-33a-5p/DKK1 in EC remains elusive.

In this study, miR-33a-5p and DKK1 were detected in serum of EC patients and EC cells to observe their clinical value and molecular mechanism in this disease, and to explore whether miR-33a-5p can affect the biological function of EC cells by targeting the DNK1-mediated Wnt/β-catenin pathway.

## RESULTS

### Clinical value of miR-33a-5p and DKK1 in EC

**Figure 1:**
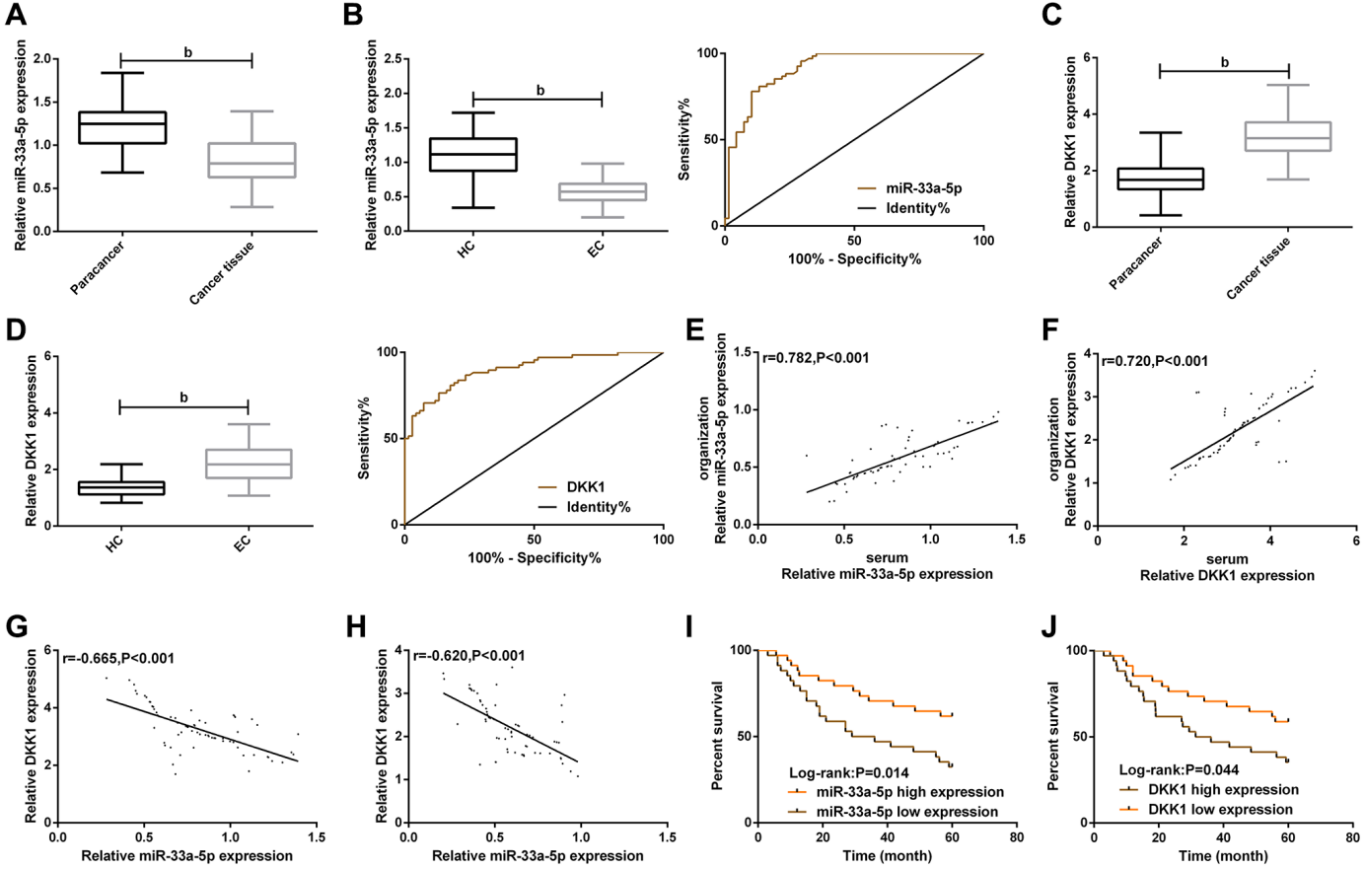
**Clinical value of miR-33a-5p and DKK1 in esophageal cancer.** (**A**) Expression of miR-33a-5p in esophageal cancer tissues; (**B**) Serum miR-33a-5p expression in esophageal cancer and ROC curve; (**C**) Expression of DKK1 in esophageal cancer; (**D**) Serum DKK1 expression in esophageal cancer and ROC curve; (**E**) Serum miR-33a-5p expression was correlated with esophageal cancer; (**F**) Serum DKK1 expression was correlated with the esophageal cancer; (**G**) Correlation between miR-33a-5p and DKK1 in esophageal cancer tissues; (**H**) Correlation between miR-33a-5p and DKK1 in serum of esophageal cancer patients; (**I**) Relationship between serum miR-33a-5p expression and 5-year OS of patients; (**J**) Relationship between serum DKK1 expression and 5-year OS of patients. The inter-group comparison was conducted by the independent sample *t*-test, mean ± SD, ^b^*P* < 0.01 for inter-group comparisons.

We initially evaluated the clinical correlation of miR-33a-5p and DKK1 with EC. Comparison of clinical data between EC patients and concurrent healthy controls showed that there was no difference between the two groups in terms of gender, age, smoking history and drinking history (Table 1). After detection, qPT-PCR showed that miR-33a-5p expression was low expressed (Figure 1A and 1B) while DKK1 expression (Figure 1C and 1D) was highly expressed in EC tissues and serum samples. The expression of miR-33a-5p or DKK1 in EC tissues was positively correlated with the expression of miR-33a-5p or DKK1 in EC serum samples, respectively (Figure 1E and 1F). Meanwhile, the expression of miR-33a-5p was negatively correlated with DKK1 expression in both EC tissues and EC serum samples (Figure 1G and 1H). ROC analysis revealed that the AUCs of serum miR-33a-5p and DKK1 for EC occurrence were 0.914 and 0.900, respectively (Table 2). Further analysis of the relationship between miR-33a-5p, DKK1 and clinical characteristics showed that tumors in most cases were larger than 5cm in size, and were mostly moderately and highly differentiated (Table 3), indicating that miR-33a-5p and DKK1 were associated with higher TNM staging and differentiation degree. Then, the correlation of miR-33a-5p and DKK1 with 5-year OS of EC patients was determined, with the corresponding median value of serum miR-33a-5p and DKK1 expression as its boundary for defining their high and low expression (Figure 1I and 1J). Survival analysis found that patients with low miR-33a-5p expression or high DKK1 expression in serum had a worse 5-year OS (Figure 1I and 1J). Cox regression analysis further indicated that TNM staging, low miR-33a-5p expression and high DKK1 expression were independent prognostic indicators of 5-year OS in patients (Table 4).

**Table 1:** Clinical data.

**Variables**	**Patients with esophageal cancer (*n* = 68)**	**Healthy controls (*n* = 68)**	**t/χ^2^**	***P***
Gender			0.736	0.392
Male	32 (47.06)	37 (54.41)		
Female	36 (52.94)	31 (45.59)		
Age			0.119	0.731
≤60 years old	32 (47.06)	30 (44.12)		
>60 years old	36 (52.94)	38 (55.88)		
History of smoking			0.538	0.464
Yes	24 (35.29)	20 (29.41)		
No	44 (64.71)	48 (70.59)		
History of drinking			0.036	0.850
Yes	20 (29.41)	19 (27.94)		
No	48 (70.59)	49 (72.06)		

**Table 2:** Occurrence value of serum miR-33a-5p and DKK1 in EC.

**Parameters**	**AUC**	**95 CI%**	**Std. Error**	**Cut-off**	**Sensitivity (%)**	**Specificity (%)**
miR-33a-5p	0.914	0.866–0.961	0.024	0.709	77.94	89.71
DKK1	0.900	0.850–0.950	0.026	1.705	76.47	86.76

Abbreviations: AUC: Area under the receiver operating characteristic curve; CI:Confidence interval.

**Table 3:** Correlation of miR-33a-5p and DKK1 with clinical characteristics of patients with EC.

**Variables**	***n***	**miR-33a-5p**	***t***	***P***	**DKK1**	***t***	***P***
Gender			0.933	0.354		0.198	0.843
Male	32	0.607 ± 0.160			2.240 ± 0.630		
Female	36	0.565 ± 0.204			2.210 ± 0.600		
Age (years)			1.520	0.133		1.017	0.313
≤60	31	0.621 ± 0.165			2.142 ± 0.601		
>60	37	0.553 ± 0.197			2.293 ± 0.616		
TNM staging			3.887	<0.001^b^		2.961	0.004^b^
I-II	33	0.663 ± 0.187			2.011 ± 0.540		
III-IV	35	0.510 ± 0.152			2.425 ± 0.610		
Tumor size (cm)			1.932	0.058		1.942	0.056
≤5	25	0.64 ± 0.215			2.040 ± 0.492		
> 5	43	0.552 ± 0.159			2.331 ± 0.650		
Vascular invasion			1.910	0.060		1.664	0.101
No	44	0.615 ± 0.188			2.135 ± 0.614		
Yes	24	0.527 ± 0.169			2.389 ± 0.579		
Differentiation degree			2.584	0.012^b^		2.426	0.018^b^
High and moderate differentiation	51	0.616 ± 0.188			2.124 ± 0.618		
Low differentiation	17	0.488 ± 0.143			2.524 ± 0.487		
Radiosensitivity			1.761	0.083		1.958	0.054
Complete/partial remission	54	0.604 ± 0.185			2.152 ± 0.578		
Ineffective	14	0.508 ± 0.170			2.503 ± 0.670		

^b^*P* < 0.01 for inter-group comparisons.

**Table 4:** Univariate and multivariate Cox regression analysis of 5-year OS in patients with EC.

**Variables**	**Univariate**		**Multivariate**	
	**HR (95 CI%)**	***P***	**HR (95 CI%)**	***P***
Gender (male & female)	1.325 (0.611–2.563)	0.482		
Age (≤60 years vs. >60 years)	1.423 (0.739–2.624)	0.275		
TNM staging (I + II vs. III + IV)	5.658 (3.097–10.287)	<0.001^b^	5.294 (2.785–9.852)	<0.001^b^
Tumor size (≤5cm vs. >5cm)	2.349 (0.897–2.038)	0.147		
Vascular invasion (no vs. yes)	0.683 (0.425–1.052)	0.091		
Differentiation degree (high/medium vs. low differentiation)	1.912 (1.275–2.758)	0.035^a^	1.108 (0.729–1.659)	0.627
Radiosensitivity (complete/partial remission vs. ineffective)	1.349 (0.705–2.547)	0.367		
DKK1 (high vs. low)	4.873 (2.615–9.463)	<0.001^b^	4.192 (2.138–8.567)	<0.001^b^
DKK1 (low vs. high)	1.915 (1.268–2.758)	0.002^b^	1.895 (1.257–2.859)	0.003^b^

^a^*P* < 0.05, ^b^*P* < 0.01 for inter-group comparisons. Abbreviations: HR: Hazard ratio; CI: Confidence interval.

### Effects of miR-33a-5p on the biological functions of EC cells

**Figure 2:**
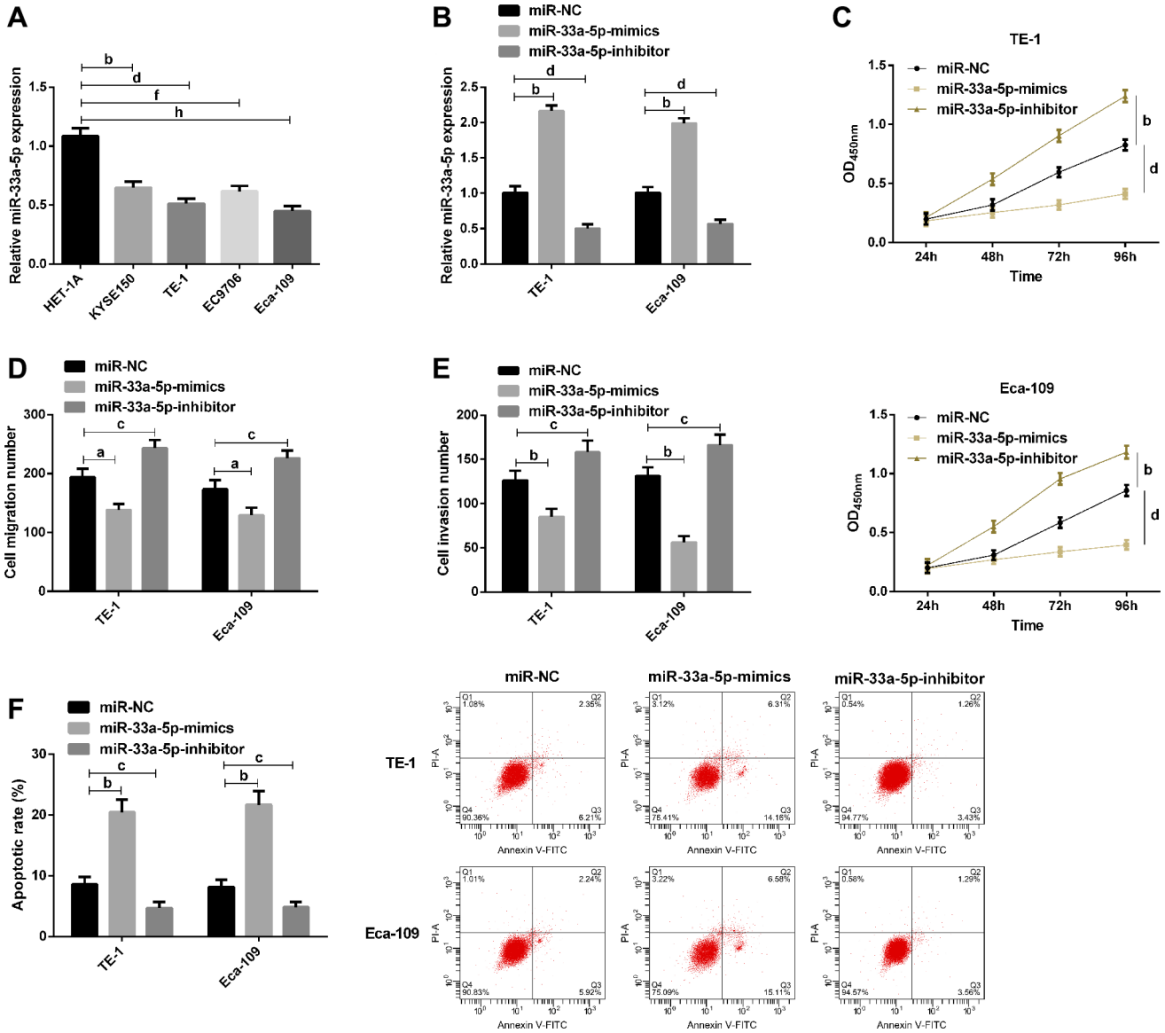
**Effects of miR-129-5p on biological function of esophageal cancer cells.** (**A**) qRT-PCR detection of miR-33a-5p expression in esophageal cancer cells by; (**B**) qRT-PCR detection of miR-33a-5p expression in TE-1, Eca-109 after transfection with miR-NC, miR-33a-5p-mimics, miR-33a-5p-inhibitor; (**C**) Cell proliferation of TE-1 and Eca-109 by CCK-8; (**D**) Migration number of TE-1 and Eca-109 cells by Transwell; (**E**) Invasion number of TE-1 and Eca-109 cells by Transwell; (**F**) Apoptosis rate of TE-1 and Eca-109 cells by flow cytometry and the apoptosis diagram. The inter-group comparison was conducted by the independent sample *t*-test, multiple time point data were analyzed by repeated measures analysis of variance (ANOVA), mean ± SD, ^b^*P* < 0.01, ^c^*P* < 0.05, ^d^*P* < 0.01, ^f^*P* < 0.01, and ^h^*P* < 0.01 for inter-group comparisons.

Then, we further explored the effect of miR-33a-5p on EC progression *in vitro*. The qRT-PCR showed miR-33a-5p expression in EC cell lines KYSE150, TE-1, EC9706, Eca-109 was lower than that in human esophageal epithelial cell line HET-1A (Figure 2A). TE-1 and Eca-109 were selected for subsequent transfection. qRT-PCR results revealed that compared with miR-NC, miR-33a-5p expression in cells up-regulated significantly after transfection with miR-33a-5p-mimics, while decreased after transfection with miR-33a-5p-inhibitor (Figure 2B). CCK-8 assay indicated that miR-33a-5p upregulation inhibited cell proliferation, while miR-33a-5p significantly enhanced cell proliferation (Figure 2C). Transwell assay results exhibited that cell migration and invasion ability were inhibited after upregulation of miR-33a-5p, but enhanced after knockdown of miR-33a-5p (Figure 2D and 2E). AnnexinV-PI double staining test results validated that miR-33a-5p overexpression induced apoptosis, while miR-33a-5p knockdown decreased the apoptotic ability of cells (Figure 2F).

### DKK1 is identified as the target of miR-33a-5p

**Figure 3:**
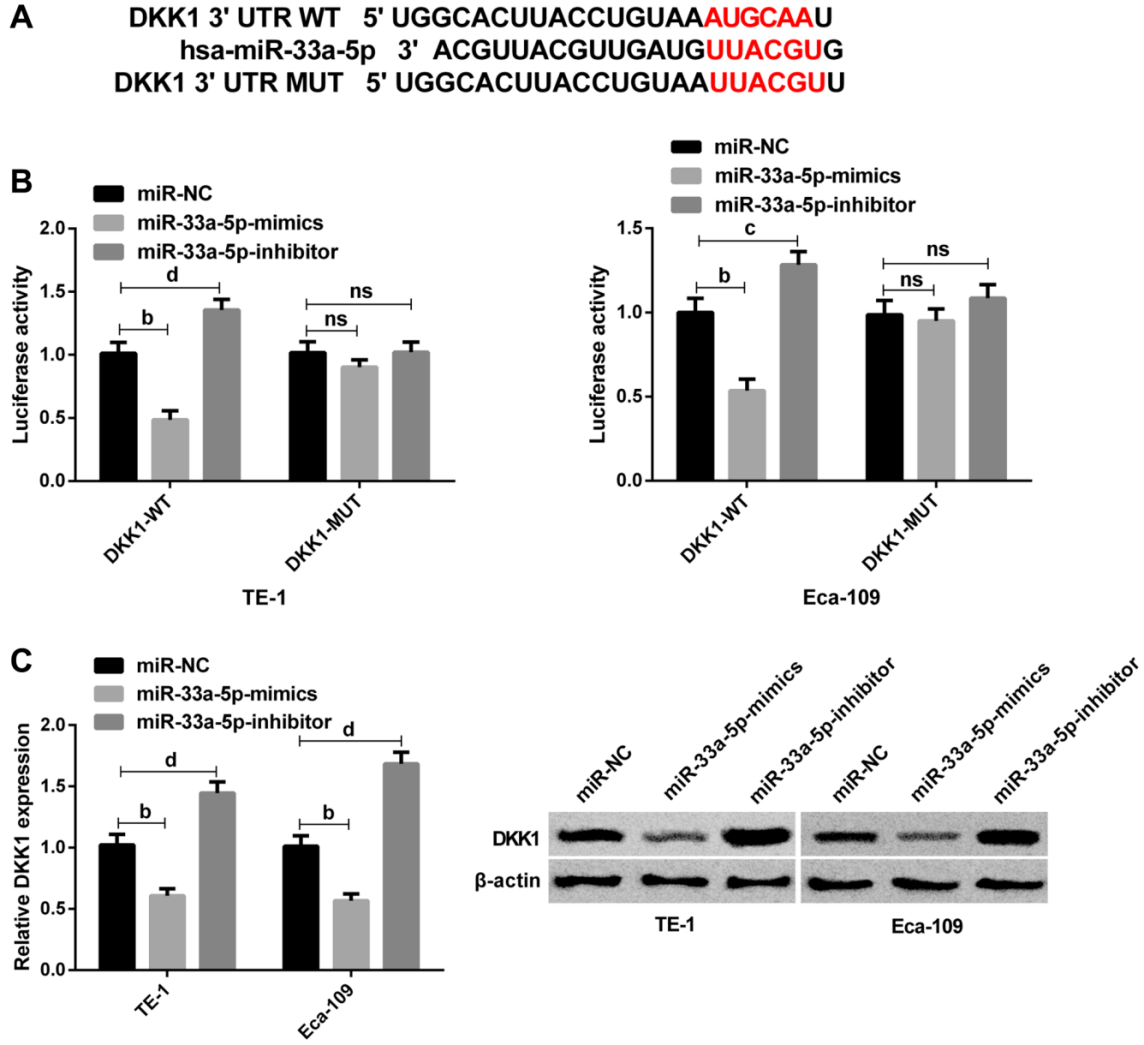
**DKK1 is identified as the target of miR-33a-5p.** (**A**) The predicted binding sites between n DKK1 and miR-33a-5p. (**B**) Interaction between DKK1 and miR-33a-5p by dual luciferase reporter assay; (**C**) Western blot of DKK1 protein expression in TE-1 and Eca-109 cells after transfection with miR-NC, miR-33a-5p-mimics and miR-33a-5p-inhibitor and the protein diagram. The inter-group comparison was conducted by the independent sample *t*-test, mean ± SD, ^b^*P* < 0.01, ^c^*P* <0.05, and ^d^*P* < 0.01 for inter-group comparisons.

Next, we explored the potential mechanism underlying miR-33a-5p-mediated EC progression. Given that miR-33a-5p could cause various biological functions of EC cells, we used TargetScan and miRDB database to predict its potential targets. Bioinformatics analysis in TargetScan and miRDB database indicated that DKK1 was the possible target of miR-33a-5p (Figure 3A). Subsequent DLR assay revealed that miR-33a-5p upregulation inhibited the activity of DKK1-WT, and conversely, miR-33a-5p downregulation significantly increased the activity of DKK1-WT; however, the luciferase activity was not affected after co-transfection with DKK1-MUT (Figure 3B). Then Western blot analysis exhibited that DKK1 protein expression was significantly reduced after transfection with miR-33a-5p-mimics, while was elevated after miR-33a-5p knockdown (Figure 3C). All these suggested that miR-33a-5p can directly regulate DKK1 expression in cells by binding to the 3′-UTR sequence.

### Effects of DKK1 on the biological behavior of EC cells

**Figure 4:**
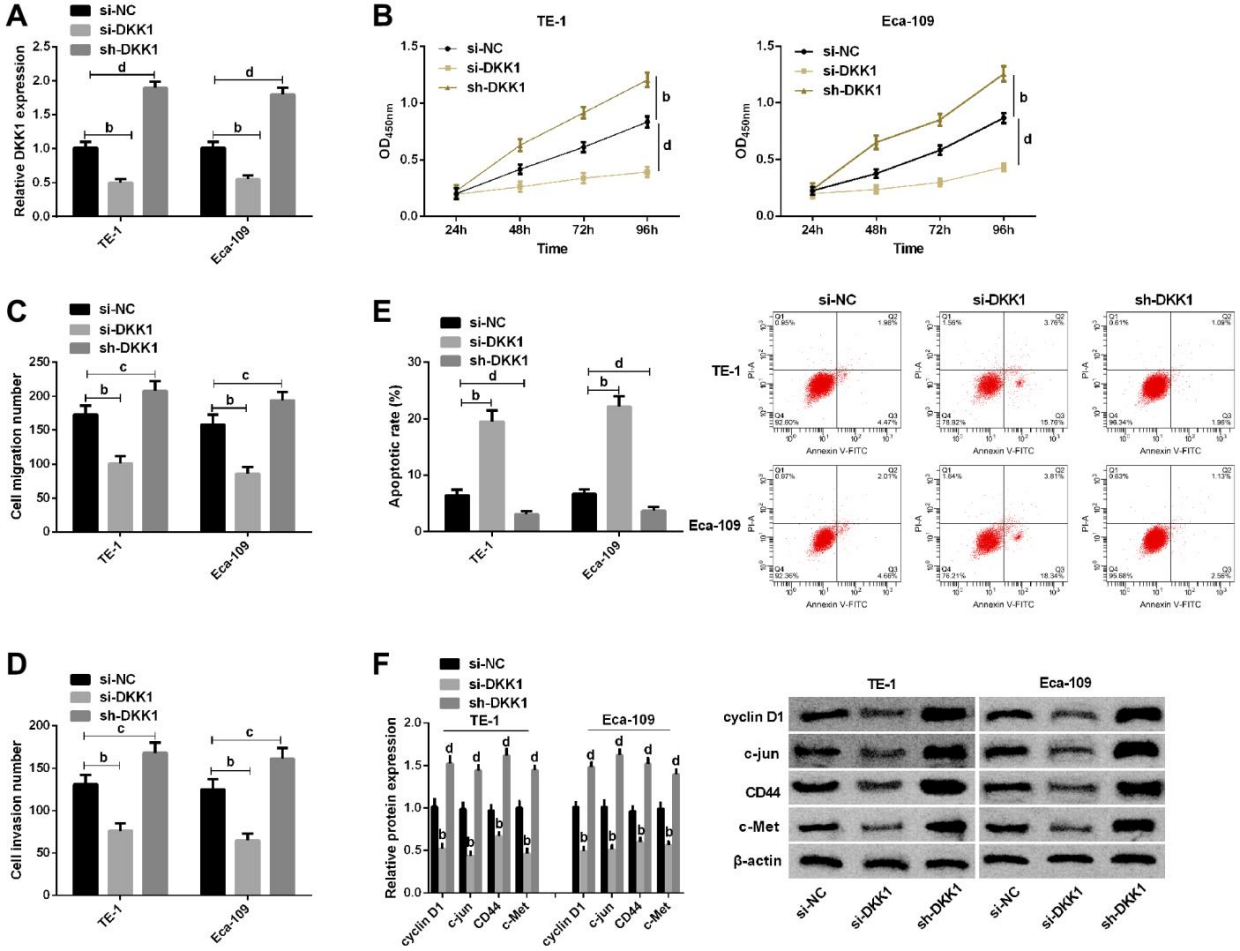
**Effects of DKK1 on biological function of esophageal cancer cells.** (**A**) qRT-PCR detection of DKK1 expression in TE-1 and Eca-109 after transfection of si-NC, si-DKK1, and sh-DKK1; (**B**) CCK-8 assay of cell proliferation of TE-1 and Eca-109; (**C**) Migration number of E-1 and Eca-109 cells by Transwell assay; (**D**) Invasion number of TE-1 and Eca-109 cells by Transwell assay; (**E**) Cells apoptosis rates of TE-1 and Eca-109 cells by Flow cytometry and the apoptosis diagram; (**F**) Western blot of Cyclin D1, c-jun, CD44 and c-Met protein expression in TE-1 and Eca-109 cells and the protein bands. The inter-group comparison was conducted by the independent sample *t*-test, multiple time point data were analyzed by repeated measures analysis of variance (ANOVA), mean ± SD, ^b^*P* < 0.01, ^c^*P* < 0.05, and ^d^*P* < 0.01 for inter-group comparisons.

Furthermore, we assessed whether DKK1 affected EC cell proliferation, migration and invasion, as well as apoptosis. The qRT-PCR showed a significant decrease in DKK1 expression after transfection with si-DKK1, while a significant increase in DKK1 expression after transfection with sh-DKK1 (Figure 4A). CCK-8 assay revealed that the proliferation of cells was inhibited after DKK1 knockdown (Figure 4B). Transwell assay exhibited that knocking down DKK1 lowered cell invasion and migration (Figure 4C and 4D). Flow cytometry analysis demonstrated that DKK1 knockdown significantly promoted cell apoptosis (Figure 4E). However, the opposite results were obtained by detecting these biological behaviors of cells after DKK1 overexpression (Figure 4E). Western blot analysis showed that knockdown of DKK1 could inhibit the expression of cyclin D1, c-jun, CD44, and c-Met proteins in cells, and correspondingly, overexpression of DKK1 can promote the expression of cyclin D1, c-jun, CD44, and c-Met proteins (Figure 4F).

### miR-33a-5p targets DKK1-mediated Wnt/β-catenin pathway to influence biological behavior of EC

**Figure 5:**
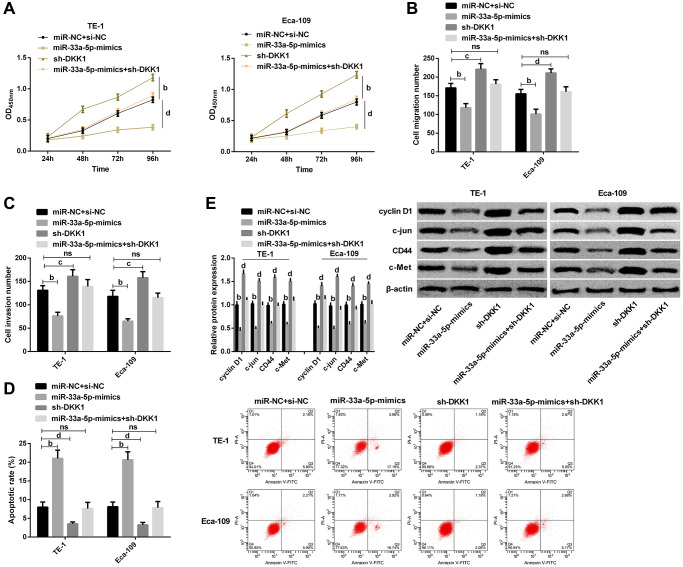
**miR-33a-5p targeted DKK1-mediated Wnt/β-catenin pathway to affect biological behavior of esophageal cancer cells.** (**A**) Cell proliferation of TE-1, Eca-109 by CCK-8; (**B**) Number of migration of TE-1 and Eca-109 cells by Transwell; (**C**) Number of invasion of TE-1 and Eca-109 cells by Transwell; (**D**) Cell apoptosis rates of TE-1and Eca-109 cells by Flow cytometry and the apoptosis map; (**E**) Western blot of Cyclin D1, c-jun, CD44, and c-Met protein expression in TE-1 and Eca-109 cells and the protein bands. The inter-group comparison was conducted by the independent sample *t*-test, multiple time point data were analyzed by repeated measures analysis of variance (ANOVA), mean ± SD, ^b^*P* < 0.01, ^c^*P* < 0.05, and ^d^*P* < 0.01 for inter-group comparisons.

To further confirm whether miR-33a-5p affects EC by regulating DKK1, we transfect TE-1, Eca-109 cells with miR-33a-5p-mimics, miR-NC + si-NC, sh-DKK1, and miR-33a-5p-mimics + sh-DKK1. It was observed that DKK1 upregulation offset the effects of miR-33a-5p overexpression on the proliferation (Figure 5A), migration (Figure 5B), invasion (Figure 5C), and apoptosis (Figure 5D) of TE-1 and Eca-109 cells. Western blot detection demonstrated that cyclin D1, c-jun, CD44, and c-Met protein expression levels were not statistically difference between cells transfected with miR-33a-5p-mimics + sh-DKK1 and those transfected with miR-NC + si-NC (Figure 5E); however, compared with sh-DKK1 transfected cells, the expression levels of cyclin D1, c-jun, CD44, and c-Met protein expression were markedly decreased in cells transfected with miR-33a-5p-mimics + sh-DKK1 (Figure 5E).

### miR-33a-5p inhibits the tumor growth of EC *in vivo*

**Figure 6:**
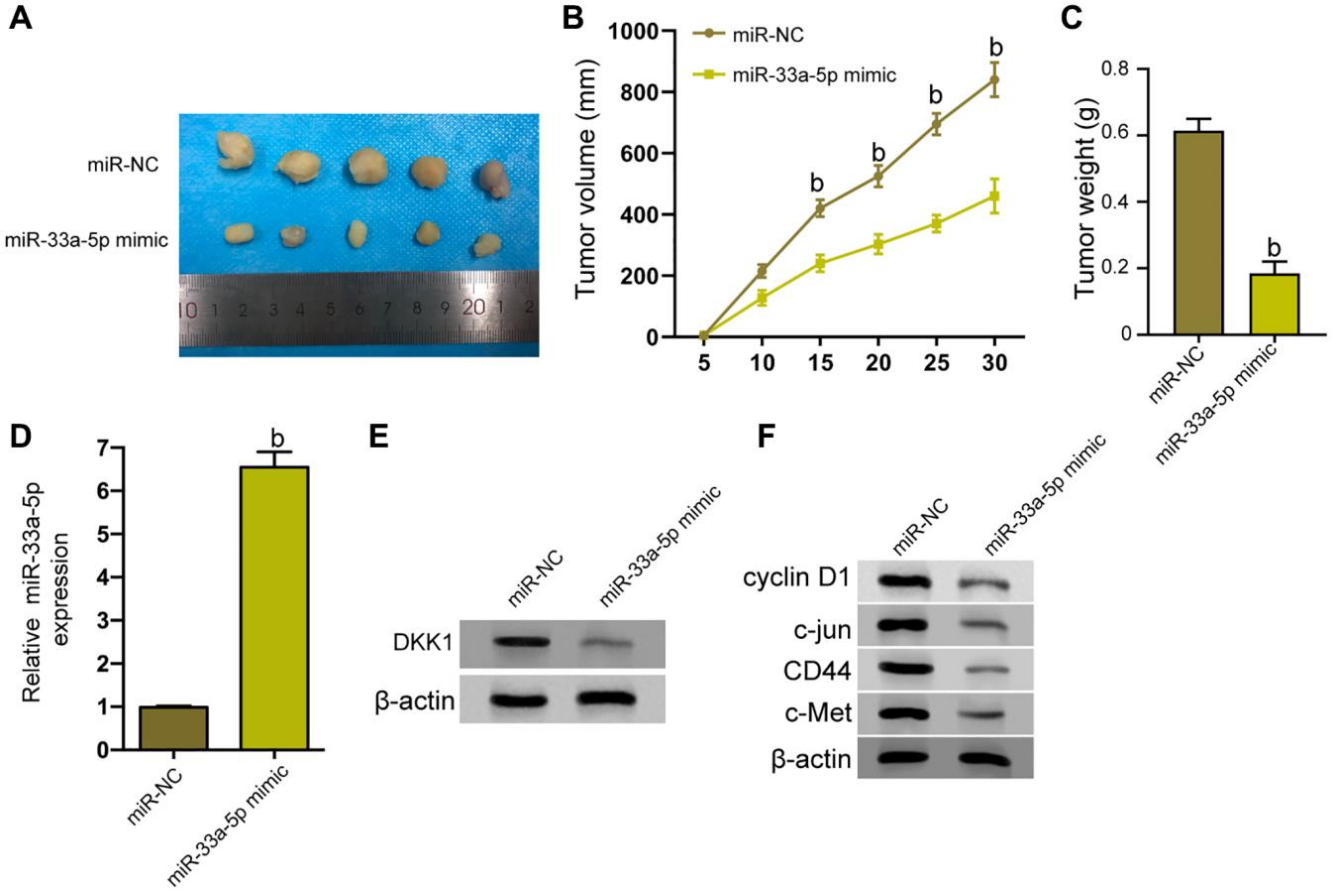
**miR-33a-5p inhibits the tumor growth of EC *in vivo*.** (**A**–**F**) The effect of miR-33a-5p on tumor growth of EC cells *in vivo* was analyzed by nude mice tumorigenicity assay by injected with the TE-1 cells treated with control mimic or miR-33a-5p mimic. (**A**) Representative images of dissected tumors from nude mice were presented. (**B**) The average tumor volume was calculated and shown. (**C**) The average tumor weight was calculated and shown. (**D**) The expression levels of miR-33a-5p were measured by qPCR in the tumor tissues of the mice. (**E**) The protein expression of DKK1 was assessed by Western blot analysis in the tumor tissues of the mice. (**F**) Western blot of Cyclin D1, c-jun, CD44, and c-Met protein expression in TE-1 and Eca-109 cells and the protein bands. *N* = 5. The inter-group comparison was conducted by the independent sample *t*-test, multiple time point data were analyzed by repeated measures analysis of variance (ANOVA), mean ± SD, ^b^*P* < 0.01.

Next, we tried to explore the impact o miR-33a-5p on the EC cell growth *in vivo*. To this end, the tumorigenicity analysis was carried out in nude mice injected with TE-1 cells treated with control mimic or miR-33a-5p-mimic. Significantly, the miR-33a-5p-mimic attenuated the tumor growth of TE-1 cells *in vivo*, as demonstrated by the decreased tumor size (Figure 6A), repressed tumor volume (Figure 6B), and reduced tumor weight (Figure 6C). As expected, the miR-33a-5p expression was up-regulated and DKK1 expression was down-regulated by miR-33a-5p-mimic in the tumor tissues of the mice (Figure 6D and 6E). Consistently, the levels of cyclin D1, c-jun, CD44, and c-Met protein were repressed by miR-33a-5p-mimic in the model (Figure 6F).

## DISCUSSION

EC is a frequent occurring and common disease in humans with a high mortality rate [16]. In this study, we found that miR-33a-5p was low expressed in EC, while DKK1 was up-regulated, both of which could be used as potential markers for EC. In addition, miR-33a-5p can target the DKK1-mediated Wnt/β-catenin pathway to affect the biological behavior of EC. Our study revealed that miR-33a-5p and DKK1 can be used as potential markers for EC, which may contribute to the research of miR in the field of EC research.

Although X-ray or endoscopy is a useful tool for the diagnosis of EC, these detection methods still have certain limitations [17]. Recent studies have shown that miRNAs play a key role in the biological processes of various tumors, and are related to tumorigenesis and development [18, 19]. In the present study, we found that miR-33a-5p can act as an inhibitor in EC, and the AUC of serum miR-33a-5p in the occurrence of EC was 0.914, which has a good value. In addition, underexpressed miR-33a-5p was associated with high TNM staging and low degree of pathological differentiation, and predicted a poor prognosis in patients, suggesting that miR-33a-5p can serve as a potential marker for EC. There is evidence reporting that miR-33a-5p is down-regulated in lung cancer tissues, cells and serum, which is a potentially effective marker of early lung cancer [20]. Although our study confirmed that miR-33a-5p can be a potentially reliable biomarker for EC, the mechanism of miR-33a-5p in EC is still unclear.

Some studies have shown that miR-33a-5p plays a role in the biological process of tumor cells, including cell proliferation, differentiation, and apoptosis [21, 22]. For example, miR-33a-5p was found to be underexpressed in lung adenocarcinoma, which could directly target the mTOR pathway *in vitro* or *in vivo* to increase sensitivity to Celastrol, and improve antitumor effects and inhibit cell proliferation capacity [23]. In tongue squamous cell carcinoma, miR-33a-5p expression was significantly underexpressed, and CASC15 can directly target miR-33a-5p to promote the growth of tongue squamous cell carcinoma cells [24]. In this study, miR-33a-5p overexpression inhibited EC cell proliferation, migration and invasion, and promoted apoptosis, while these biological functions were reversed after miR-33a-5p knockdown, suggesting that miR-33a-5p is expected to be a potential therapeutic target for EC. But how it is involved in these biological functions remains unknown. We predicted that DKK1 may be the target of miR-33a-5p through the database, which were later verified by DLR assay. Previous studies have shown that DKK1 expression is up-regulated in solid tumors such as hepatocellular carcinoma [25] and ESCC [26]. In the present study, DKK1 acted as an oncogenic factor in EC, and the AUC for EC occurrence was 0.900; moreover, increased DKK1 expression indicated a more aggressive EC and a worse prognosis. Similarly, Yamabuki et al. [27] showed that serum DKK1 was highly expressed in ESCC and lung cancer, and might be a diagnostic and prognostic marker of these diseases. Our research also revealed that the malignant phenotype of EC cells can be inhibited by knocking out DKK1. On the contrary, overexpression of DKK1 can enhance the malignant phenotype of EC cells. We can thus preliminarily conclude that miR-33a-5p can directly target DKK1, a direct target of miR-33a-5p, to regulate the proliferation, migration, invasion and apoptosis of EC.

Abnormal activation of the Wnt/β-catenin pathway has been found to play an important role in the occurrence and development of tumors, and its pathway-related factors cyclin D1, c-jun, CD44, and c-Met are considered to be effective oncogenes in tumor progression [28, 29]. According to previous studies, miR-33a-5p can be regulated after PNMA1 transcription and promote progression of hepatocyte cancer cells by activating the Wnt/β-catenin pathway [30]. Other evidence has shown that, by targeting DKK1, miR-501-5p can activate the Wnt/β-catenin pathway in gastric cancer cells to participate in tumor progression [31]. In this study, DKK1 knockdown lead to markedly reduced expression levels of cyclin D1, c-jun, CD44, and c-Met proteins, and conversely, DKK1 overexpression activated the Wnt/β-catenin pathway. The up-regulation of DKK1 eliminated the effects of miR-33a-5p overexpression on proliferation, migration, invasion and apoptosis of EC cells, as well as the Wnt/β-catenin signaling pathway related factors, suggesting that miR-33a-5p upregulation can directly target the DKK1-mediated Wnt/β-catenin pathway to inhibit the malignant biological properties of EC cells. However, there are still some shortcomings in this study. We only conducted *in vitro* experiments without *in vivo* experiments to observe the tumor inhibitory effect of miR-33a-5p. In regards with the size of the tumour, most of the cases were more than 5 cm which possibly means more extended disease, so it is easier to identify miR-33a-5p, DKK1 levels in the serum or tissue. Also, the differentiation of tumors is mainly high and moderate with only a few cases of low differentiation, which might also play a role in the expression levels of miR-33a-5p and DKK1. Thus, the underlying mechanisms need to be further explored. Most patients had tumor size > 5cm may indicate the EC patients in this study were all in the late stage of cancer. Hence, the clinical significance of miR-33a-5 to identify early stage of EC remained unknown. Moreover, due to the small number of cases, the diagnosis and clinical value of serum miR-33a-5p and DKK1 in esophageal cancer still need to be further verified by clinical practice. The role of miR-33a-5p and DKKl/Wnt signaling in cancer development has been demonstrated and the mechanism of miR-33a-5p inhibits EC progression needs to be further developed and deepened. Other potential mechanisms should be explored in future instigations.

In conclusion, the upregulation of miR-33a-5p can directly target the DKK1-mediated Wnt/β-catenin pathway to inhibit the proliferation, invasion and migration of EC cells and induces apoptosis, suggesting that miR-33a-5p is a candidate biomarker for EC.

## MATERIALS AND METHODS

### Clinical data

In total 68 patients with EC admitted and treated in Tumor Hospital of Liaocheng from March 2012 to March 2014 were selected. Inclusion criteria: Diagnosed as EC by pathological examination, all the patients met the TNM staging criteria issued by AJCC in 2017 [32] and had not received any prior treatment before surgery, with complete clinical data. Exclusion criteria: Patients with other malignant tumors, severe liver and kidney dysfunction, or infection were excluded, as well as those who lost to follow up; Patients with other diseases which might lead to the abnormal increase or decrease of miR-33a-5p or DKKl were excluded. Additionally, 68 healthy controls were selected. With the approval of the Medical Ethics Committee of Tumor Hospital of Liaocheng and the written informed consent obtained from all the participants, this study was conducted. EC patients were followed up for 5 years, and the total survival (OS) was from the time of surgery to the end of follow-up or the patient’s death [33].

### Sample collection

Elbow vein blood (5 ml) was collected from EC patients (before surgery) and healthy controls and centrifuged at 4°C and 800g for 10 min. Healthy controls didn't have EC or any other type of cancer. The resulting supernatant was then transferred to the test tube for cryopreservation. After surgical resection, specimens of paired EC and normal tissues (5 cm from the edge of the tumor) were collected, frozen in liquid nitrogen tank, and then stored in a cryogenic refrigerator at -80°C for use.

### qRT-PCR detection

miRNA was extracted from serum by miRNeasy kit (Invitrogen, Carlsbad, CA, USA, AM1561), while the RNA from cells and tissues was extracted using the TRIzol kit (Biolab Technology Co., Ltd., Beijing, China). The purity and the concentration of RNA were determined by SMA6000 microspectrophotometer (Merinton Instrument Co., Ltd., Beijing, China). For the determination of miR, the total RNA was reverse transcribed into cDNA using the TaqMan miRNA reverse transcription kit (Biolab Technology Co., Ltd., Beijing, China) according to the kit instructions, and PCR quantification was performed with the TaqMan Universal PCR Master Mix kit (Chundu Biotechnology, Co., Ltd., Wuhan, China). As to the determination of mRNA, reverse transcription of total RNA into cDNA was conducted by Prime Script RT kit (Future Biotechnology, Co., Ltd., Beijing, China), and PCR quantification was carried out using the SYBR Green qPCR superMix-UDG kit (Biolab Technology Co., Ltd., Beijing, China). On an ABI 7300 real-time PCR system (ThermoFisher Scientific, Co., Ltd., Shanghai, China), Then qPCR was performed on the ABI 7300 real-time PCR system (Thermo Fisher Scientific, Shanghai, China). The test was repeated 3 times. U6 and GAPDH were used as internal controls of miR-33a-5p and GAPDH respectively, and the expression levels of miR-33a-5p and GAPDH were calculated using 2^−ΔΔCt^. The primers were synthesized and purchased (GenScript, China). Primer sequences: miR-33a-5p: Forward: 5′-GTGCATTGTAGTTGCATTGCA-3′; Reverse: 5′-GTGCAGGGTCCGAGGT-3′. DKK1: Forward: 5′-CCTTGAACTCGGTTCTCAATTCC-3′; Reverse: 5′-CAATGGTCTGGTACTTATTCCCG-3′. U6: Forward: 5′-GCTTCGGCAGCACATATACTAAAAT-3′; Reverse: 5′-CGCTTCACGAATTTGCGTGTCAT-3′. GAPDH: Forward: 5′-ATTCCATGGCACCGTCAAGGCTGA-3′, Reverse: 5′-TTCTCCATGGTGGTGAAGACGCCA-3′.

### Cell culture and transfection

EC cell lines KYSE150, TE-1, EC9706 and Eca-109, as well as human esophageal epithelial cell line HET-1A (Otwo Biotechnology Co., Ltd., Shenzhen, China) were all cultured in Roswell Park Memorial Institute (RPMI1640) medium (Biolab Technology Co., Ltd., Beijing, China) containing 10% fetal bovine serum (FBS, Gibco, NY, USA) and penicillin/streptomycin double antibodies (SolarBio, China) in a 37°C and 5% CO_2_ constant temperature cell incubator. Transfection was performed when the confluence reached 70%. miR-33a-5p-mimics, miR-335-3-inhibitor, miR-33a-5p negative control sequence (miR-NC), DKK1 RNA negative control (si-NC), targeted inhibition of DKK1 RNA (si-DKK1) and targeted overexpression of DKK1 RNA (sh- DKK1) were synthesized and obtained from ThermoFisher Scientific, Shanghai, China. The transfection process was carried out in strict accordance with the instructions of Lipofectamine 3000 transfection reagent (Biolab Technology Co., Ltd., Beijing, China).

### Detection of cell proliferation

The cell proliferation test was carried out according to the instructions of the cell counting kit-8 (CCK-8; Yiyan Biotechnology Co., Ltd., Shanghai, China). Cells were seeded into 96-well plates. The absorbance (OD) values of the cells were measured at specific time points. CCK-8 solution (10 μL) was added to each well for another 2-hour culture at the time of detection, and then the OD value was measured at the 450nm wavelength on a AMR-100 automatic microplate reader (Allsheng Instrument Co., Ltd., Hangzhou, China).

### AnnexinV-PI double staining for apoptosis detection

The collected cells were digested with trypsin and resuspended with 200 μL binding buffer. Then, 5 μL AnnexinV-FITC (Beyotime, China) was added to each well and incubated at 4°C in dark for 15 min before the addition of 10 μL PI solution. After incubation for 5 min, CytoFLEX flow cytometry (Beckman Coulter Commercial Enterprise (China), Co., Ltd.) was applied to detect cell apoptosis.

### Transwell chamber assay for cell migration and invasion

Migration detection: 24 hours after transfection, cells were seeded into the Transwell upper chamber (Corning, NY, USA) at 5 × 10^5^ cells/well and supplemented with serum-free Dulbecco's modified eagle medium (DMEM) to 200 μL, while 700 μL DMEM containing 10% FBS was added to the lower chamber for culture in a 37°C and 5% CO_2_ thermostatic cell incubator.

Twenty-four hours later, the chamber was taken out, and the cells remaining in the upper chamber were cleared away. Then the cells were immobilized with formaldehyde for 10 min and dyed with crystal violet for 30 min at room temperature. Finally, cell counting was performed under a microscope, with 5 fields being randomly observed in each hole, and the number of cell transmembrane was compared. Invasion detection: serum-free DMEM and Matrigel glue were used to prepare Matrigel diluent at 8:1. Then the diluent was added to the bottom of the upper chamber at 50 μL per well and placed overnight at 4°C. The follow-up experiment steps were the same as the migration assay.

### Western blot detection

After cell lysis, bicinchoninic acid (BCA) (Abbkine, CA, USA) method was used to detect the protein concentration, and 30 μL protein was separated using 12% sodium dodecyl sulfate- polyacrylamide gel electrophoresis (SDS-PAGE). Then the protein was transferred to a Polyvinylidene Fluoride (PVDF) membrane (Millipore, MA, USA), sealed with 5% skim milk powder for 2 h, and added with cyclin D1, c-jun, CD44, c-Met and β-actin primary antibodies purchased from Shanghai Beyotime Biotechnology Co., Ltd. (Dilution ratio: 1: 1000, AF1183, AF1612, AF0105, AF1432, AF0003), for overnight incubation at 4°C. Then TBST buffer was applied for three rinses of the membrane, 10min each, followed by a 3-minute incubation with the corresponding horseradish peroxidase-labeled goat anti-rabbit (HRP)-labeled secondary antibody (Beyotime Biotechnology Co., Ltd., Shanghai, China, dilution ratio: 1:1000, A0208). The membrane was finally developed by ECL (Millipore, Germany), and the grayscale values were analyzed using Quantity One 1-D software.

### Dual luciferase reporter (DLR) assay

TargetScan and miRDB database [34] predicted that DKK1 was a possible target of miR-33a-5p. The wild type sequences of the 3′UTR region of DKK1 were cloned into the pmirGLO vectors and purchased (GeneScript, China). The site-specific mutated sequences (shown in Figure 3A) of the 3′UTR of DKK1 were inserted into pmirGLO vectors and purchased (GenScript, China). Lipofectamine 3000 was used to co-transfect DKK1-WT or DKK1-MUT with miR-33a-5p-mimic, miR-33a-5p-inhibitor or miR-NC into cells. Forty-eight hours after transfection, DLR gene assay kit (Shanghai Beinuo Biotechnology Co., Ltd., China, SLDL-100) was utilized to measure luciferase activity.

### Xenograft tumor model

All animal experiments in this study were approved by the Tumor Hospital of Liaocheng. BALB/c nude mice aged 5 weeks were purchased from Shanghai Laboratory Animal Center of China. TE-1 cells were transfected with miR-33a-5p mimic or negative control, and suspended in saline at a density of 1×10^7^ cells/ml. A total of 200 μL TE-1 cell suspension was subcutaneously injected to left back of mice. The mice were euthanized at day 30, and the tumors were isolated, weighted and processed for subsequent analysis. Tumor size was measured every 5 days and calculated using the following formula: width^2^ × length/2.

### Statistical analysis

Data in line with normal distribution were expressed as mean ± SD, and the inter-group comparison was conducted by the independent sample *t*-test. Receiver operating characteristic curve (ROC) was employed to evaluate the correlation of miR-33a-5p and DKK1 with EC occurrence, and the area under the curve (AUC) was calculated. Pearson test was utilized for correlation analysis between miR-33a-5p and DKK1. Multiple time point data were analyzed by repeated measures analysis of variance (ANOVA), and Bonferroni method was used for post hoc test. Data comparison among multiple groups was performed by one-way ANOVA, and the post-hoc test was performed by LSD-t. The survival curve of patients was visualized using Kaplan-Meier method, and Log-rank was used to evaluate the difference of survival time in patients with high or low expression of miR-33a-5p and DKK1. Univariate and multivariate Cox regression analysis was used to analyze the prognostic factors of patients. A statistically significant difference was assumed at *P* < 0.05. SPSS 20.0 and GraphPad Prism 8.0 (IBM Corp, Armonk, NY, USA) were used for statistical analysis.
